# Primate Lentiviruses Modulate NF-κB Activity by Multiple Mechanisms to Fine-Tune Viral and Cellular Gene Expression

**DOI:** 10.3389/fmicb.2017.00198

**Published:** 2017-02-14

**Authors:** Elena Heusinger, Frank Kirchhoff

**Affiliations:** Institute of Molecular Virology, Ulm University Medical CenterUlm, Germany

**Keywords:** HIV, SIV, NF-κB, Nef, Vpu, Tat, LTR

## Abstract

The transcription factor nuclear factor kappa-light-chain-enhancer of activated B cells (NF-κB) plays a complex role during the replication of primate lentiviruses. On the one hand, NF-κB is essential for induction of efficient proviral gene expression. On the other hand, this transcription factor contributes to the innate immune response and induces expression of numerous cellular antiviral genes. Recent data suggest that primate lentiviruses cope with this challenge by boosting NF-κB activity early during the replication cycle to initiate Tat-driven viral transcription and suppressing it at later stages to minimize antiviral gene expression. Human and simian immunodeficiency viruses (HIV and SIV, respectively) initially exploit their accessory Nef protein to increase the responsiveness of infected CD4^+^ T cells to stimulation. Increased NF-κB activity initiates Tat expression and productive replication. These events happen quickly after infection since Nef is rapidly expressed at high levels. Later during infection, Nef proteins of HIV-2 and most SIVs exert a very different effect: by down-modulating the CD3 receptor, an essential factor for T cell receptor (TCR) signaling, they prevent stimulation of CD4^+^ T cells via antigen-presenting cells and hence suppress further induction of NF-κB and an effective antiviral immune response. Efficient LTR-driven viral transcription is maintained because it is largely independent of NF-κB in the presence of Tat. In contrast, human immunodeficiency virus type 1 (HIV-1) and its simian precursors have lost the CD3 down-modulation function of Nef and use the late viral protein U (Vpu) to inhibit NF-κB activity by suppressing its nuclear translocation. In this review, we discuss how HIV-1 and other primate lentiviruses might balance viral and antiviral gene expression through a tight temporal regulation of NF-κB activity throughout their replication cycle.

## Introduction

To allow efficient viral gene expression, replication, and spread, viral pathogens need to exploit the cellular transcriptional machinery. In some cases, they hijack exactly those transcription factors that are activated by the host response to infection to initiate antiviral immune responses. The interaction of human immunodeficiency virus type 1 (HIV-1) and related simian immunodeficiency viruses (SIVs) with the NF-κB (nuclear factor kappa-light-chain-enhancer of activated B cells) family of transcription factors provides a prime example for the hostile takeover of a key mediator of the immune response by viral pathogens (Hiscott et al., 2001; Chan and Greene, 2012). NF-κB is ubiquitously expressed and its dysregulation is associated with many pathologies including cancer, cardiovascular, pulmonary, and inflammatory diseases (Panday et al., 2016). NF-κB can be induced by multiple stimuli and regulates the expression of cellular genes involved in numerous processes, such as cell proliferation, DNA repair and cell differentiation or survival (Ghosh and Hayden, 2012; Napetschnig and Wu, 2013). Furthermore, NF-κB plays a key role in inflammation and the induction of innate and adaptive immune responses, including expression of interferon-stimulated genes (ISGs) representing a first line of defense against viral pathogens (Hayden et al., 2006; Vallabhapurapu and Karin, 2009; Pfeffer, 2011).

NF-κB has a complex role in HIV-1 replication and pathogenesis. ISGs exert numerous effector functions and may target almost every step of the retroviral replication cycle (Kluge et al., 2015; Colomer-Lluch et al., 2016). Thus, their induction by NF-κB transcription factors might have beneficial effects for the host by suppressing HIV-1 replication. However, induction of interferon and other cytokines also contributes to HIV-1-induced chronic and systemic inflammation that drives progression to AIDS. Thus, whether induction of interferon responses is beneficial or harmful in HIV-1 infection is a matter of debate (Doyle et al., 2015; Utay and Douek, 2016). Importantly, NF-κB is also critical for potent viral gene expression from the HIV-1 long terminal repeat (LTR) promoter that typically contains two adjacent NF-κB binding sites in its main enhancer region (Chan and Greene, 2012). Combinations of stimulatory drugs including NF-κB inducing agents, such as prostratin, are currently examined for their ability to activate the latent reservoirs of HIV-1 to render them susceptible for elimination (Jiang and Dandekar, 2015; Cary et al., 2016). Thus, NF-κB presents a target for therapeutic intervention both by suppressing its activation to prevent harmful inflammation (Chan and Greene, 2012) or by activating this transcription factor to stimulate the latent reservoirs of HIV-1 (Jiang and Dandekar, 2015).

Numerous studies have examined the effects of HIV-1 infection on NF-κB activity (reviewed in Chan and Greene, 2012). Altogether, the results were puzzling and frequently controversial. Recent evidence suggests that HIV-1 and other primate lentiviruses may have differential effects during the early and late stages of their replication cycle and tightly regulate NF-κB activity (Sauter et al., 2015). In the present review, we first briefly describe some basic aspects of NF-κB signaling and its interaction with the LTR promoter of HIV-1 and other primate lentiviruses. Subsequently, we discuss how these viruses might modulate NF-κB activity throughout their replication cycle to ensure efficient viral gene expression while minimizing the expression of antiviral genes.

## Basic Mechanisms of NF-κB Signaling

NF-κB was initially discovered in the laboratory of David Baltimore because of its ability to bind the enhancer region of the immunoglobulin κ light-chain- in B cells (Sen and Baltimore, 1986). Since then, five mammalian NF-κB members, i.e., NF-κB1 (p50/p105), NF-κB2 (p52/p100), RelA (p65), RelB and c-Rel, have been identified (Nabel and Verma, 1993). All of them share a highly conserved N-terminal Rel homology domain that is critical for dimerization and DNA binding, while three of them (p65, RelB, and c-Rel) contain an additional C-terminal transactivation domain. The remaining two factors (NF-κB1 and NF-κB2) are synthesized as large inactive precursors (p105 and p100) that need to undergo proteasomal degradation of their ankyrin repeat containing C-terminal region to generate the mature p50 and p52 NF-κB subunits, respectively (Karin and Ben-Neriah, 2000). DNA binding and transcriptional regulation requires dimerization of two subunits, with p50/p65 dimers being the most abundant active form. Many other dimer combinations were observed but not all of them act as transcriptional activators; e.g., p50/p50 and p52/p52 homodimers were reported to suppress NF-κB mediated transcription as they lack a transactivation domain (Zhong et al., 2002).

**FIGURE 1:**
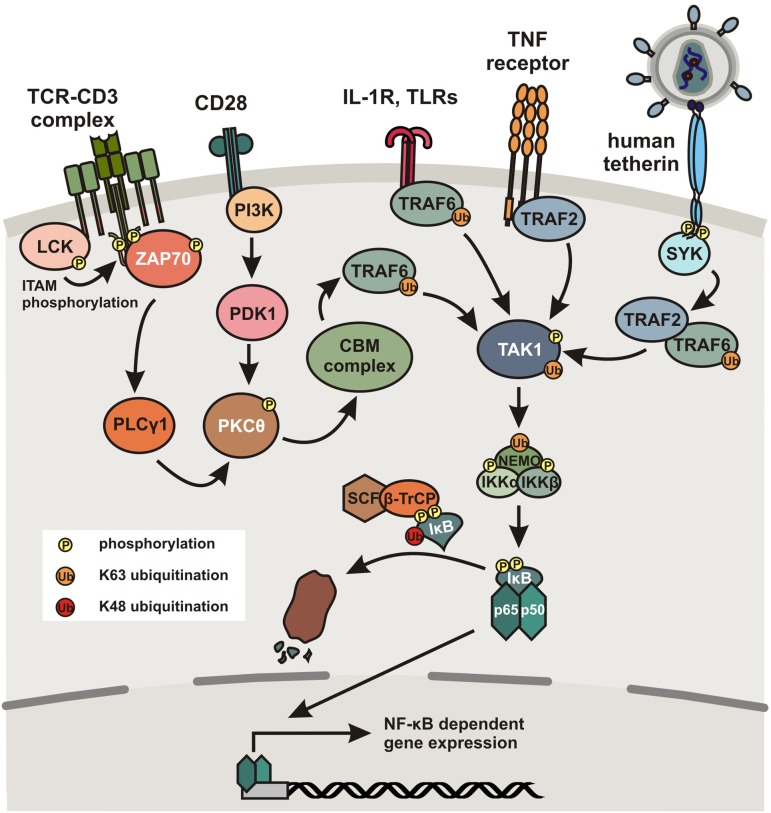
**Activation of the canonical NF-κB pathway.** Simplified schematic presentation of NF-κB activation by different cellular receptors. Binding of the TCR-CD3 complex to antigens presented by MHC molecules induces LCK-dependent phosphorylation of ITAMs in the cytoplasmic tail of CD3. This in turn recruits ZAP70, which induces a signal cascade involving activation of PLCγ1 and PKC𝜃. Alternatively, PKC𝜃 can also be activated via a signal cascade including PI3K following binding of the TCR costimulatory factor CD28 to a B7 ligand. PKC𝜃 subsequently activates the CBM complex consisting of CARD11, BCL10 and MALT1, which activates TAK1 in a TRAF6 dependent manner. Ligand binding to TLRs or TNF receptors also induces TAK1 activation in a TRAF6- or TRAF2-dependent manner, respectively. In humans (and to a much lesser extent in chimpanzees), the cellular restriction factor tetherin also acquired the ability to activate NF-κB. Upon tethering of newly formed viral particles and clustering of tetherin dimers, the cytoplasmic tail of tetherin becomes phosphorylated at a conserved YxY motif by a Scr-family kinase in a RICH2-dependent manner, recruiting SYK, which initiates a signaling cascade involving TRAF2 and/or TRAF6 resulting in the activation of TAK1. TAK1 subsequently phosphorylates IKKβ at two serine residues in the activation loop to activate IKK. Furthermore, TRAFs activate NEMO by poly-ubiquitination, followed by activation of the catalytic subunits IKKα and IKKβ. Subsequently, IκB is phosphorylated and poly-ubiquitinated by the SCF/β-TrCP complex, which results in its proteasomal degradation, thereby releasing p50/p65 heterodimers, which translocate to the nucleus where they bind to specific κB binding sites and initiate NF-κB-dependent gene expression.

In unstimulated cells, NF-κB dimers are bound to so-called inhibitors of κB (IκBs) containing ankyrin repeats that mask nuclear localization signals (NLS) and thereby keep the NF-κB proteins sequestered in the cytoplasm. The p105 and p100 precursors also contain ankyrin repeats in their C-terminal parts. Thus, they inhibit NF-κB activity and may also be classified as IκB proteins. A multitude of stimuli including viral antigens, immune cells, mitogens and cytokines, specific cell surface receptors, such as the T cell receptor (TCR)-CD3 complex, IFN receptors, the interleukin 1 receptor (IL-1R), Toll-like receptors (TLRs), or tumor necrosis factor (TNF) receptors induce degradation of IκB proteins (Vallabhapurapu and Karin, 2009). The main mechanism (canonical pathway) involves activation of IκB kinase (IKK), a heterodimer composed of the catalytic IKKα and IKKβ subunits and a regulatory factor termed NEMO (NF-κB essential modulator), and subsequent phosphorylation of serine residues in IκB regulatory domains allowing its ubiquitination and proteasomal degradation (**Figure 1**). Degradation of IκB allows exposure of the NLS and thus entry of the NF-κB complex into the nucleus for DNA binding and transcriptional induction of genes containing the appropriate binding sites within their promoter regions. As outlined below, NF-κB activation induces immune responses as well as HIV-1 proviral gene expression. Notably, NF-κB also induces expression of IκBα, resulting in a negative autoregulatory feedback loop and thus oscillating levels of NF-κB activity (Nelson et al., 2004). More in depth reviews of NF-κB signaling and its regulation are provided by Oeckinghaus and Ghosh (2009), Skaug et al. (2009) and Vallabhapurapu and Karin (2009).

## Role of NF-κB in Immunity and Inflammation

It is well established that NF-κB transcription factors regulate many genes involved in immune and inflammatory responses (reviewed in Vallabhapurapu and Karin, 2009). Upon pathogen recognition, a variety of cellular pattern recognition receptors (PRRs), including cGAS [Cyclic-GMP-AMP (cGAMP) synthase], STING (stimulator of interferon genes), IFI16 (interferon γ-inducible protein 16), RIG-I-like helicases, NOD-like receptors (NLRs) and TLRs may activate the NF-κB pathway. Thus, NF-κB signaling is induced by multiple stimuli, including pathogen associated molecular patterns (PAMPs) as well as proinflammatory cytokines, e.g., tumor necrosis factor-α (TNF-α) and interleukin-1. Ligand binding to TLRs induces several downstream effectors and results in the formation of signaling complexes activating the IKK complex (**Figure 1**). Ubiquitination of TNF-receptor-associated factors (TRAF) and NEMO facilitates activation of the catalytic IKKβ subunit resulting in the phosphorylation and proteasomal degradation of IκB and consequently nuclear translocation of active NF-κB dimers (Skaug et al., 2009). Similarly, signaling via members of the TNF receptor superfamily ultimately leads to activation of the IKK complex, following recruitment of various adaptor proteins and stimulation of TGF-β-activated kinase 1 (TAK1; Wang and Baldwin, 1998). Furthermore, some activated TNFR superfamily members may induce accumulation of mitogen-activated protein kinase 14 (MAP3K14 or NF-κB-inducing kinase, NIK) that activates IKKα to induce processing of p100 and thus activation of e.g., RelB/p52 heterodimers to mediate expression of NF-κB-responsive genes via the non-canonical pathway. This pathway is slower than the canonical NF-κB signaling pathway and can also be activated via the CD40 ligand (CD40L), receptor activator of NF-κB ligand (RANKL) or lymphotoxin β (Cildir et al., 2016). Following activation, RelB/p52 heterodimers translocate to the nucleus for target gene activation.

NF-κB is not only a key factor in the induction of effective innate immune responses but also plays an important role in adaptive immunity. For example, ligand binding to the TCR complex induces recruitment of LCK tyrosine kinase that phosphorylates the ITAMs of the CD3 ζ chains (**Figure 1**). Subsequent recruitment of ZAP70 mediates activation of a signaling pathway involving PLCγ, protein kinase C (PKC) family members, the CARD11-BCL10-MALT1 (CBM) complex, TRAF6, and ultimately TAK1, inducing NF-κB activity by phosphorylation and activation of IKKs (Cheng et al., 2011; Paul and Schaefer, 2013). NF-κB activation is further enhanced by costimulation via CD28, which triggers distinct signaling cascades involving PI3K and PDK1 (Boomer and Green, 2010). Thus, the signaling pathways induced by CD3/CD28-mediated stimulation of T cells upon interaction with antigen-presenting cells (APCs) allow NF-κB to enter the nucleus to upregulate expression of cytokines and antimicrobial effectors as well as genes involved in T cell survival, proliferation, and differentiation. Consequently, regulation of NF-κB activity plays a key role in innate and adaptive immune function and the defense against bacterial and viral pathogens. In agreement with this important role in inflammatory gene expression, chronic activation of NF-κB signaling is observed in a variety of inflammatory diseases including arthritis, sepsis, gastritis, asthma, atherosclerosis, and inflammatory bowel disease (Panday et al., 2016).

## Role of NF-κB in Primate Lentiviral Transcription

**FIGURE 2:**
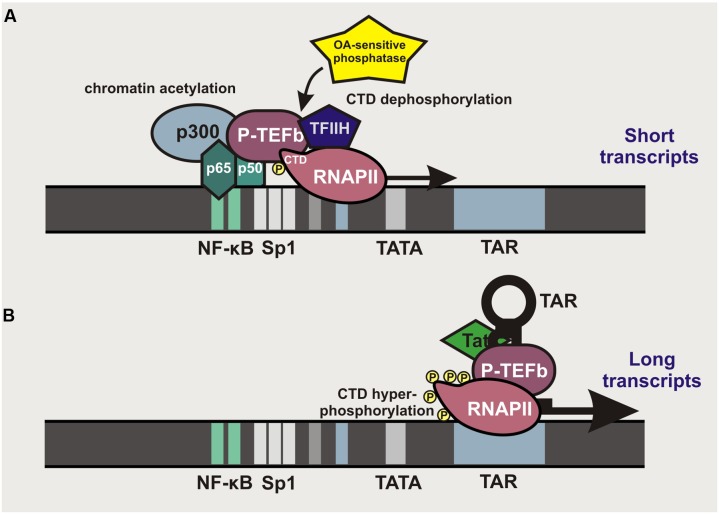
**Role of κB binding sites in primate lentiviral LTR-mediated transcription. (A)** Localization of κB binding sites in primate lentiviral LTRs. NF-κB binding allows recruitment of p300 to initiate chromatin acetylation and to render the LTR better accessible for RNAPII. NF-κB also recruits P-TEFb, which binds to the CTD of RNAPII and strongly enhances its processivity. De-phosphorylation of the CTD by an OA-sensitive phosphatase terminates the elongation, resulting in the production of short TAR-containing transcripts. **(B)** In the presence of the viral Tat protein, transcription of proviral DNA is maintained independently of NF-κB. Tat binds to the short hairpin loop of TAR and recruits P-TEFb to the RNAPII, thereby allowing efficient elongation and generation of full-length viral transcripts.

It is long known that HIV-1 transcription can be stimulated by activation of the canonical NF-κB pathway (Nabel and Baltimore, 1987; Tong-Starksen et al., 1987). NF-κB binding sites are found in the enhancer region of all primate lentiviral LTRs, although their numbers may vary between different subtypes of HIV-1 group M and various groups of SIV and HIV. Most subtypes of pandemic HIV-1 group M strains (A, B, D, F, G, H, J, and K) and some SIVs contain two NF-κB binding sites located –104 to –80 bp upstream of the transcriptional start site within the 5′LTR (**Figure 2**). In contrast, the second human immunodeficiency virus (HIV-2), subtype A/E recombinants of HIV-1 group M and several SIV lineages contain just a single NF-κB binding site. Finally, subtype C strains, which account for almost 50% of HIV-1 infections worldwide, typically contain three binding sites for NF-κB in their enhancer region (Bachu et al., 2012). LTR-mediated transcription is initiated by binding of p50/p65 heterodimers or other members of the NF-κB transcription factor family that recruit p300 to promote acetylation of LTR chromatin and allow access for RNA polymerase II (RNAPII; **Figure 2A**). NF-κB binding also promotes transcriptional elongation by recruiting the positive transcription elongation factor b (P-TEFb) complex and the transcription factor TFIIH to the carboxyl domain (CTD) of RNAPII, allowing its phosphorylation that results in increased processivity of this enzyme (Barboric et al., 2001). However, this effect is transient since an okadaic acid (OA)-sensitive phosphatase dephosphorylates the CTD of RNAPII to prevent P-TEFb interaction. Thus, short transcripts terminated shortly after the trans-activation response (TAR) RNA element predominate at the early stage. Accumulation of the viral Tat protein that binds to TAR and recruits P-TEFb allows the virus to overcome this bottleneck by allowing hyper-phosphorylation of RNAPII and potent transcriptional elongation and the generation of full-length viral transcripts (Williams et al., 2007) (**Figure 2B**).

Typically, mutation of the NF-κB binding sites in HIV-1 LTRs will prevent efficient proviral transcription. However, one case of a replication-competent pathogenic HIV-1 strain lacking NF-κB binding sites has been reported (Zhang et al., 1997). This unusual HIV-1 strain contained duplications in the TCF-1alpha region that may have compensatory effects. Thus, NF-κB binding sites are important but not essential for HIV-1 replication or pathogenicity *in vivo*. Since NF-κB activation stimulates HIV-1 transcription, it is also targeted in approaches aiming to eliminate the latent viral reservoirs. Induction of NF-κB activity by T cell activation or treatment with phorbol esters, such as prostratin, potently enhance viral gene expression (Coudronniere et al., 2000; Williams et al., 2004). However, since NF-κB transcription factors are involved in numerous physiological and pathological processes, their induction is prone to adverse effects. Notably, some members of the NF-κB transcription factor family may also promote HIV-1 latency, i.e., it has been reported that in unstimulated T cells, p50 homodimers occupy the NF-κB sites in the proviral LTR to recruit HDAC1, thereby promoting histone hypo-acetylation and hence chromatin condensation rendering the viral LTR poorly accessible to RNAPII binding (Williams et al., 2006).

## Induction of NF-κB Signaling by Viral Immune Sensing and DNA Damage Responses

HIV-1 infection modulates NF-κB signaling by multiple mechanisms. As outlined above, sensing of viral PAMPs by PRRs activates NF-κB to induce an antiviral immune response. HIV-1 sensing is not fully understood as the virus has evolved effective evasion mechanisms. For example, recent evidence suggests that the viral capsid stays largely intact until it docks to the nucleopore (Arhel et al., 2007; Lelek et al., 2012; Dharan et al., 2016) and that it recruits cellular factors, such as PSF6 and cyclophilins, to prevent innate immune activation (Rasaiyaah et al., 2013). Nonetheless, it has become clear that a variety of viral components may trigger immune responses. Viral RNA or DNA intermediates of the reverse transcription (RT) process can be sensed by cytosolic receptors, such as cGAS, IFI16, PQBP1 and RIG-I, particularly under suboptimal conditions for efficient RT (Jakobsen et al., 2015; Sauter and Kirchhoff, 2016). Moreover, antiretroviral restriction factors may also act as immune sensors (Hotter et al., 2013). For example, TRIM5α induces untimely uncoating of the viral capsid and may also act as an activator of the TAK1 kinase complex to stimulate AP-1 and NF-κB signaling (Pertel et al., 2011). Similarly, trapping of HIV-1 particles by the host restriction factor tetherin induces phosphorylation of tyrosine residues in its cytoplasmic tail to mediate recruitment of SYK tyrosine kinase and TRAF2 and/or 6 to activate TAK1 and consequently NF-κB-dependent immune responses (Galão et al., 2012, 2014; Tokarev et al., 2013) (**Figure 1**). Interestingly, this sensing function of tetherin seems to have an evolutionary recent origin and is only observed for the human and (to a much lesser extent) chimpanzee orthologs (Galão et al., 2012), whereas the ability of tetherin to block virion release has a very ancient origin (Heusinger et al., 2015; Blanco-Melo et al., 2016). Finally, HIV-1 infection induces DNA damage responses since the generation of linear viral RNA/DNA and DNA species, non-integrated circular forms of viral DNA and a double-stand break in the host-genome are inevitable concomitants of the viral replication cycle. DNA damage signaling may activate ATM and mediate phosphorylation and ubiquitination of NEMO to induce IKK and consequently NF-κB activation (Miyamoto, 2011; McCool and Miyamoto, 2012).

## Activation of NF-κB to Initiate Early Viral Gene Transcription

HIV-1 not only affects NF-κB activation by triggering immune sensors and inducing DNA damage responses but may also use some of its gene products to manipulate this transcription factor to promote efficient viral gene expression. Many studies investigated the effect of HIV-1 on NF-κB activation but the initial results were often contradictory. One possible reason for this is that NF-κB signaling plays a complex role in the viral replication cycle and that effects may depend on the cell type and state of activation, as well as the stimuli and HIV-1 strains or proteins used in the respective studies. Furthermore, accumulating evidence suggests that the HIV-1 Nef and Tat proteins that are expressed at high levels immediately after initiation of proviral transcription enhance and late viral gene products, such as Vpu, suppress NF-κB activation (Roux et al., 2000; Akari et al., 2001; Bour et al., 2001; Varin et al., 2005; Herbein et al., 2008; Mangino et al., 2011; Fiume et al., 2012; Liu et al., 2013). It has been reported that Tat interacts with IκBα and the p65 subunit of NF-κB to prevent binding of the repressor to the NF-κB complex while promoting p65 binding to DNA (Fiume et al., 2012). It has also been shown that the cytoplasmic domain of the HIV-1 envelope glycoprotein (Env) gp41 interacts with TAK1 to induce NF-κB activation (Postler and Desrosiers, 2012). Whether this mechanism is effective during the earliest stage of infection, i.e., induced by virion fusion with the plasma cell membrane of the target cell, or requires higher quantities of Env achieved during later stages of the replication cycle remains to be determined. Finally, a variety of studies reported that Vpr modulates NF-κB signaling in various cell types. However, the effects are controversial and both stimulatory and inhibitory effects of Vpr have been described (Kogan et al., 2013; Liu et al., 2013, 2014; Liang et al., 2015). Thus, the effects of virion-associated and *de novo* synthesized Vpr in primary HIV-1 infected T cells need to be further investigated.

**FIGURE 3:**
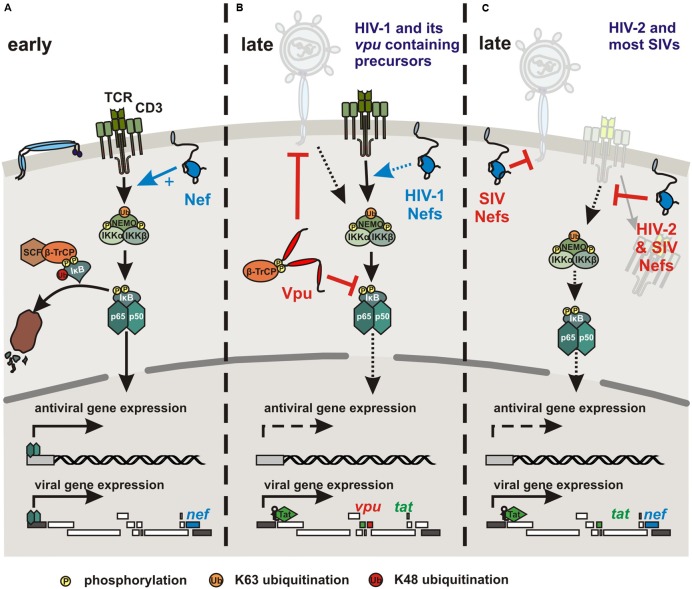
**Modulation of NF-κB activity by HIV and SIV Nef and Vpu proteins. (A)** To initiate early viral gene expression, the viral accessory protein Nef promotes NF-κB activation by boosting TCR-CD3 mediated T cell activation and other yet to be determined mechanisms. **(B)** HIV-1 and its closest SIV counterparts use their Vpu protein to inhibit NF-κB and thus antiviral gene expression during late stages of the replication cycle. Vpu interferes with IκB degradation by sequestration of β-TrCP and other as-yet-unknown mechanisms. Furthermore, HIV-1 group M and (less effectively) N Vpu proteins counteract the cellular restriction factor tetherin, which traps budding virions at the cell surface and also acts as NF-κB activating immune sensor in the case of the human ortholog. In the presence of the viral transactivator Tat, viral transcription is maintained independently of NF-κB activity. **(C)** HIV-2 and most SIV strains do not contain a *vpu* gene but express Nef proteins that efficiently down-modulate CD3 from the cell surface to prevent T cell activation and hence to suppress the induction of NF-κB and other transcription factors. SIV Nefs also counteract tetherin in their respective host. However, although monkey tetherins restrict virus release they are not known to act as NF-κB activating immune sensors.

The accessory viral Nef protein does not induce NF-κB activity on its own but boosts the responsiveness of HIV-1 infected cells to stimulation (Alexander et al., 1997; Simmons et al., 2001; Fenard et al., 2005) (**Figure 3A**). Nef-mediated activation of NF-κB, nuclear factor of activated T cells (NFAT), IL-2 and LTR stimulation following TCR-CD3/CD28 costimulation seems to require association with lipid rafts (Schrager and Marsh, 1999; Fortin et al., 2004; Kumar et al., 2016) and may depend on the state of activation of infected T cells (Baur et al., 1994). Interactions of HIV-1 Nef with the CD3 ζ chain (Xu et al., 1999) and downstream effectors of TCR signaling, such as the tyrosine kinase LCK (Baur et al., 1997), serine/threonine p21-activating kinases (Sawai et al., 1994), the DOCK2-ELMO1 complex (Janardhan et al., 2004) and ERK/MAP kinases (Schrager et al., 2002) have been reported. Thus, Nef might affect the catalytic activity of different kinases, induce cytoskeletal changes, and activate a variety of signaling pathways. The relative contribution of these activities and interactions to Nef-mediated enhancement of T cell activation is largely unclear. In either case, Nef promotes nuclear translocation of NF-κB and other transcription factors, such as AP1 and NFAT and activates the viral promoter to induce Tat expression and hence productive viral replication (Kinoshita et al., 1998). Notably, Nef may exert its multiple functions rapidly after viral entry since it is expressed at high levels early during the viral replication cycle and possibly even before proviral integration (Sloan et al., 2011).

## Late Inhibition of NF-κB Signaling by HIV-1 and its *Vpu* Containing SIV Counterparts

While Nef-mediated enhancement of NF-κB activity may be important to initiate proviral transcription, it is less critical after accumulation of the viral Tat protein and may even become detrimental to HIV-1 replication because of the induction of antiviral gene expression. Thus, HIV-1 and other primate lentiviruses might tightly regulate NF-κB activity throughout their replication cycle to allow proviral transcription while minimizing antiviral gene expression. Recent studies show that the accessory viral protein U (Vpu) potently suppresses NF-κB activity during later stages of the viral replication cycle (**Figure 3B**) (Akari et al., 2001; Bour et al., 2001; Sauter et al., 2015). A *vpu* gene was most likely acquired by the precursor of SIVs infecting *Cercopithecus* monkeys with subsequent cross-species transmissions and recombination events giving rise to other *vpu* containing primate lentiviruses (Bailes et al., 2003; Takeuchi et al., 2015). Thus, *vpu* is found in HIV-1, its chimpanzee and gorilla precursors, SIVcpz and SIVgor, and in SIVgsn, SIVmus, SIVmon, and SIVden, infecting greater spot-nosed, mustached, mona, and Dent’s mona monkeys, respectively (Kirchhoff, 2009). The Vpu proteins of pandemic HIV-1 group M strains counteract tetherin-mediated activation of NF-κB-dependent antiviral immune responses (Cocka and Bates, 2012; Galão et al., 2012, 2014; Tokarev et al., 2013). This is expected since the Vpus of HIV-1 group M and SIVs infecting several *Cercopithecus* species are potent antagonists of tetherin-mediated inhibition of virion release. More surprisingly, Vpus derived from SIVs infecting chimpanzees and gorillas or HIV-1 group O strains that use their Nef protein to counteract tetherin are also potent inhibitors of NF-κB (Sauter et al., 2015). Indeed, early studies suggested that HIV-1 Vpu prevents NF-κB activation by inhibiting degradation of IκB through sequestration of the adaptor protein β-TrCP (Akari et al., 2001; Bour et al., 2001). More recent data show that the ability of Vpu to prevent NF-κB activation independently of the stimulus is conserved between all lineages of SIV and HIV-1 (except group N) containing this accessory gene and does not correlate with β-TrCP interaction or tetherin antagonism (Sauter et al., 2015). In agreement with the results of early studies (Bour et al., 2001), an intact β-TrCP binding motif and interaction of Vpu with β-TrCP are essential for highly effective inhibition of p65 nuclear translocation by primary Vpu proteins. However, some Vpus failing to recruit β-TrCP still suppressed NF-κB-dependent gene expression (Sauter et al., 2015). Thus, the action of Vpu involves stabilization of IκB and reduced nuclear translocation of p65 but also additional yet-to-be-defined mechanisms. Importantly, the ability of Vpu to inhibit NF-κB is dominant over the stimulatory effect of Nef and associated with reduced induction of IFN and ISGs in HIV-1 infected T cells (Sauter et al., 2015). Accordingly, HIV-1 and its closest simian counterparts seem to utilize Nef to boost NF-κB activation to initiate LTR-driven proviral transcription. At later stages, when viral gene expression is ensured by the presence of Tat, Vpu inhibits NF-κB activity to limit expression of antiviral genes and to attenuate the immune response.

## Potential Suppression of NF-κB Activation by Nef-Mediated Down-Modulation of TCR-CD3

The ability to inhibit NF-κB activity is highly conserved among primate lentiviral Vpu proteins (Sauter et al., 2015) suggesting an important role for viral immune evasion *in vivo*. As outlined above, however, *vpu* genes are only found in HIV-1 and its closest simian counterparts, raising the question whether the majority of primate lentiviruses use another mechanism to inhibit NF-κB during late stages of the replication cycle. It is tempting to speculate that originally essentially all primate lentiviruses used the CD3 down-modulation function of Nef to suppress the activity of NF-κB and other transcription factors. This Nef function is conserved among HIV-2 and most lineages of SIVs but was entirely lost in the great majority of *vpu* containing primate lentiviruses (Schindler et al., 2006). This was most likely not just coincidence. In fact, phylogenetic and functional analyses suggest that the CD3 down-modulation function of Nef may have been lost twice during primate lentiviral evolution when the virus acquired a *vpu* gene. The first time after acquisition of *vpu* by a precursor of SIVs nowadays found in various *Cercopithecus* monkeys and a second time when this virus recombined with the precursor of SIVrcm from Red-capped mangabeys in chimpanzees to give rise to SIVcpz, the precursor of HIV-1 (Kirchhoff, 2010).

It has been suggested that Vpu might diminish the selective advantage of Nef-mediated CD3 down-modulation because it counteracts tetherin and potentially other antiviral factors induced in an inflammatory environment. In fact, lack of CD3 down-modulation by Nef is associated with higher expression levels of early (CD69) and late (CD25) activation markers as well as increased levels of apoptosis, induction of Fas, Fas-L, PD-1, and CTLA-4 cell surface expression and secretion of interferon gamma (IFN-γ) in virally infected cultures of peripheral blood mononuclear cells (Schindler et al., 2006; Schmökel et al., 2011; Yu et al., 2015). The consequences of the presence of *vpu* and the inability of Nef to block TCR-mediated T cell activation, that distinguish HIV-1 and its simian precursors from other primate lentiviruses, for viral pathogenicity largely remain a matter of speculation. While it is evident that host properties play an important role in the clinical outcome of primate lentiviral infections (Chahroudi et al., 2012), it is conceivable that potent inhibition of T cell activation should help to prevent damagingly high levels of immune activation that drive CD4^+^ T cell depletion and progression to immunodeficiency (Sodora et al., 2009). Indeed, inefficient Nef-mediated down-modulation of CD3 correlates with low numbers of CD4^+^ T cells in SIVsmm infected sooty mangabeys (Schindler et al., 2008) and viremic HIV-2 infected individuals (Khalid et al., 2012). Furthermore, HIV-1 is more pathogenic than HIV-2 (Nyamweya et al., 2013) and many SIVs do not cause disease in their natural host species (Chahroudi et al., 2012), whereas SIVcpz causes AIDS in chimpanzees (Keele et al., 2009). It is unknown whether other *vpu* containing viruses cause disease in the wild. However, the prevalence of SIVgsn/mus/mon in their natural simian hosts seems to be much lower (∼1–4%) than of SIVs capable of CD3 down-modulation (often >40%; Heigele et al., 2016). It will be interesting to further examine whether this is due to differences in the pathogenic outcome of these infections.

Vpu may allow primate lentiviruses to better cope with the antiviral immune response not only by antagonizing innate antiviral factors but also by downregulation of various receptors involved in the activation of natural killer cells (Sugden et al., 2016). However, the recent finding that Vpu suppresses NF-κB activity also suggests a more direct link with the CD3 down-modulation function of Nef. As described above, signaling via the TCR-CD3 complex induces a cascade of events ultimately leading to IKK activation and translocation of NF-κB into the nucleus for target gene expression (**Figure 1**). It has been established that Nef-mediated down-modulation of CD3 potently blocks the responsiveness of virally infected T cells to TCR-mediated stimulation (Iafrate et al., 1997; Bell et al., 1998; Khalid et al., 2012) and prevents the formation of the immunological synapse between virally infected primary CD4^+^ T cells and dendritic cells or macrophages (Arhel et al., 2009). It has been shown that CD3 down-modulation potently inhibits the induction of NFAT (Khalid et al., 2012), which also plays an important role in the immune response (Müller and Rao, 2010). While the effect on NF-κB activity is less well investigated, preliminary data clearly indicate that Nef-mediated down-modulation of CD3 would also block activation of this transcription factor (**Figure 3C**). Thus, our current knowledge suggests that most primate lentiviruses may prevent NF-κB activation by Nef-mediated down-modulation of CD3, whereas HIV-1 and its simian precursors utilize Vpu to inhibit NF-κB signaling further downstream in the cascade. The former mechanism is associated with a more “resting” phenotype of virally infected T cells and disrupts their interaction with and responsiveness to other immune cells (Arhel et al., 2009). The latter may still allow activation of the infected T cells e.g., by APCs but prevent NF-κB-dependent antiviral gene expression during later stages of the viral replication cycle. Notably, most SIVs use their Nef protein to counteract tetherin (Jia et al., 2009; Sauter et al., 2009; Zhang et al., 2009). However, this does most likely not affect NF-κB activity since only the human but not monkey orthologs of tetherin are known to activate this transcription factor (Galão et al., 2012). Finally, HIV-2 uses Env to counteract restriction by human tetherin (Le Tortorec and Neil, 2009) but it has not been reported whether this mechanism also suppresses induction of NF-κB activity.

Importantly, the effects of all primate lentiviral Nefs on T cell activation may differ during the early and late stages of the viral replication cycle. Perhaps most notably, Nef proteins from HIV-2 and SIVs that down-modulate CD3 enhance IKKβ-induced NF-κB activation as efficiently as HIV-1 or SIV Nefs lacking the CD3 down-modulation function entirely (Sauter et al., 2015). Thus, primate lentiviral Nef proteins may generally boost the responsiveness to stimulation during the earliest stage of infection. While down-modulation of CD3 from the cell surface by Nef proteins possessing this function is highly effective, it may take more time than providing an initial boost to NF-κB activity to initiate viral gene expression and productive replication. Consequently, early stimulation and late inhibition of NF-κB may both be achieved by most primate lentiviruses and be mediated by cooperative Nef and Vpu functions in HIV-1 and its precursors. Finally, it is noteworthy that even HIV-1 Nef might have differential effects depending on the stage of infection. It is puzzling that for HIV-1 Nef enhancing (Herbein et al., 2008; Mangino et al., 2011), inhibitory (Niederman et al., 1992; Bandres and Ratner, 1994), and no (Yoon and Kim, 1999; Witte et al., 2008) effects on NF-κB activity have been reported. In part, these differences may depend on the state of activation of infected T cells. However, it will also be interesting to further examine whether the effect of HIV-1 Nef might differ at different stages of the viral replication cycle. In agreement with this possibility, it is known that HIV-1 Nefs down-modulate CD28, an important costimulatory factor of T cell activation, albeit substantially less effectively than HIV-2 and most SIV Nefs (Bell et al., 2001; Swigut et al., 2001). Timing adds another degree of complexity to the already complex role of the multi-functional Nef protein throughout the viral life cycle and clearly warrants further examination.

## Conclusion and Perspectives

Numerous studies have investigated how accessory proteins of HIV and SIV, i.e., Vif, Vpr, Nef, Vpu and/or Vpx, counteract antiviral restriction factors, such as APOBEC proteins, tetherin, SAMHD1 and SERINC3/5. Expression of most of these and other antiviral factors is induced by a very limited number of transcription factors that are activated upon viral immune sensing including NF-κB and IRFs. Modulation of the induction and activity of these key regulators of innate and adaptive immunity may have a major benefit for viral replication but has only recently gained significant scientific attention. Specifically, recent data provide evidence that primate lentiviruses tightly regulate the activity of NF-κB to initiate efficient viral transcription while minimizing the expression of antiviral genes. As outlined above, the most common mechanism amongst primate lentiviruses may be initial boosting of NF-κB activity by Nef and prevention of further stimulation of T cell activation by potent down-modulation of TCR-CD3. This strategy seems to be highly successful considering the high prevalence of these viruses and the benign relationship with their natural simian hosts. In contrast, HIV-1 and its SIV precursors seem to use Nef to initially boost and Vpu to later on suppress NF-κB activation. A variety of Vpu functions that might provide a selective advance for viral immune evasion and replication have been reported (Sandberg et al., 2012; Sugden et al., 2016). Nonetheless, the emergence of a *vpu* containing subset of primate lentiviruses is somewhat surprising as they seem to be less prevalent and potentially more virulent in their natural hosts than other SIVs. Perhaps they have advantages in specific hosts as supported by the more efficient spread of HIV-1 in the human population compared to HIV-2 despite higher virulence. Notably, a few primate lentiviruses lack both *vpu* and the CD3 down-modulation function of Nef (Schmökel et al., 2011) and our preliminary data suggest that they might use yet another accessory protein (i.e., Vpr) to suppress NF-κB activity during the late stages of infection.

Altogether, recent evidence suggests that primate lentiviruses have evolved several sophisticated mechanisms to tightly regulate the activity of NF-κB and possibly other transcription factors throughout their replication cycle. The conservation of these functions supports an important role for viral replication and immune evasion. Furthermore, modulation of NF-κB activity is most likely also relevant for viral latency and inflammatory responses. NF-κB is targeted for the treatment of inflammatory and proliferative diseases as well as cancer and activation of the latent reservoirs of HIV-1 (Jiang and Dandekar, 2015; Panday et al., 2016). Because of its important role in many physiological processes, however, therapeutic modulation of NF-κB activity is prone to undesired adverse effects. Nevertheless, further studies on the mechanisms used by HIV-1 and other primate lentiviruses to manipulate this central transcription factor may provide important information on how these viruses establish latency and induce inflammation and perhaps even on how to prevent this.

## Author Contributions

EH and FK both wrote the manuscript and prepared figures.

## Conflict of Interest Statement

The authors declare that the research was conducted in the absence of any commercial or financial relationships that could be construed as a potential conflict of interest.
